# Chemically-Induced DNA Damage, Mutagenesis, and Cancer

**DOI:** 10.3390/ijms19061767

**Published:** 2018-06-14

**Authors:** Ashis K. Basu, Takehiko Nohmi

**Affiliations:** 1 Department of Chemistry, University of Connecticut, Storrs, CT 06269, USA; 2 Biological Safety Research Center, National Institute of Health Sciences, 3-25-26 Tonomachi, Kawasaki-ku, Kawasaki-shi, Kanagawa 210-9501, Japan

A large fraction of human cancers arise from exposure to chemicals, although radiation, oxidation, and genetic factors play critical roles as well. DNA damage by these agents in a cell is an important first step in the process of carcinogenesis. DNA repair processes have evolved to repair these damages. However, replication of damaged DNA may occur frequently prior to repair, resulting in gene mutations and generation of altered proteins. Mutations in an oncogene, a tumor-suppressor gene, or a gene that controls the cell cycle give rise to a clonal cell population with an advantage in proliferation. The complex process of carcinogenesis includes many such events, but has been generally considered to be comprised of the three main stages known as initiation, promotion, and progression, which ultimately give rise to the induction of human cancer. A well-documented example of carcinogenesis is the long-term use of tobacco, which radically increases the risk of lung cancer. Fifteen articles published in this Special Issue entitled “Chemically-Induced DNA Damage, Mutagenesis, and Cancer” provide an overview on the topic of the “consequence of DNA damage” in the context of human cancer with their challenges and highlights.

The topics of the published articles cover a wide range, which include the study of DNA adducts, DNA polymerase, DNA repair and repair defects, nanoparticle-induced DNA damage and cell death, metal toxicity, apoptosis, risk of melanoma, chemoprevention, radiosensitizer for primary culture tumor cells, signaling of a tumor suppressor gene, and adjuvant for chemotherapy. 

A mini-review on this topic described the history and discussed mechanistic details of the process of carcinogenesis as it gradually unfolds to reveal the steps of initiation, promotion, and progression, leading to cancer [1]. It also summarized the somatic mutation theory and the hallmarks of cancer. 

In a perspective on DNA adduct studies, Villalta and Balbo discussed the future of DNA adductomic analyses [2]. They anticipate that DNA adductomics will become a standard method to investigate covalent modification of DNA and that mass spectrometry, which has seen tremendous growth in recent years, will be used to provide highly reliable and sensitive data. 

In a review of the DNA repair protein *O*^6^-alkylguanine DNA alkyltransferase (OGT), Miggiano et al. reported recent studies on two prokaryotic OGTs, from the pathogenic bacterium *Mycobacterium tuberculosis* and the hyperthermophilic archaeon *Sulfolobus solfataricus* [3]. The role of OGTs in the biology of these microorganisms was revealed, and the authors conferred the general properties of this class of proteins. In another DNA repair related article, Kakehashi et al. investigated the role of deficiency of 8-oxoguanine glycosylase 1 (Ogg1), which repairs the 8-oxo-7,8-dihydro-2′-deoxyguanosine (8-oxodGuo) residues in DNA, using the multiorgan carcinogenesis bioassay in mice [4]. They show increased susceptibility of Ogg1 mutant mice to the multiorgan carcinogenesis induced by five different alkylating agents. The authors believe that this bioassay will be helpful to examine the influence of various targets on mouse carcinogenesis.

Boldinova et al. wrote a review on PrimPol, a human DNA polymerase with primase activity, which also is involved in DNA damage tolerance, prevention of genome instability, and mitochondrial DNA maintenance [5]. The authors discussed recent advances in biochemical and crystallographic studies of PrimPol and provided a list of their protein–protein interaction partners. Additionally, possible functions of PrimPol in both the nucleus and the mitochondria were discussed.

Singh et al. reviewed how exposure to engineered nanomaterials (ENM) might influence DNA repair pathways [6]. They focused on the differential regulation of DNA repair pathways, triggered by various ENMs. The factors that dictate aberrant repair processes, including intracellular signaling, spatial interactions, and ENM-specific responses, were discussed. In another nanoparticle work, Cellai et al. studied Fe_3_O_4_ nanoparticles in the presence or absence of an alternating magnetic field (AMF) of 186 kHz [7]. The levels of oxidative damage (measured by the levels of M_1_dG (3-(2-deoxy-*β*-d-erythro-pentofuranosyl)pyrimido[1,2-*α*]purin-10(3*H*)-one) and 8-oxodGuo, the biomarkers for lipid peroxidation and/or oxidative damage) increased considerably after 24 h incubations relative to controls. The oxidative DNA damage reached a steady-state following treatment with 60 μg/mL Fe_3_O_4_-NPs. Adduct formation increased after magnetic hyperthermia, with the highest amounts of oxidative lesions generated after a 40-min exposure to AMF. The effects of magnetic hyperthermia were considerably enhanced with increased exposure and incubation times. Notably, the levels of oxidative lesions in AMF-exposed NP-treated cells were up to 20-fold greater relative to those observed in nonexposed NP treated cells. The authors conclude that generation of oxidative lesions may be a mechanism by which magnetic hyperthermia induces cancer cell death.

Ko et al. evaluated the role of resveratrol, a polyphenol that possesses anti-oxidant, anti-inflammatory, cardioprotective, and anti-cancer properties, in cancer therapy [8]. The focus of this review is resveratrol’s in vivo and in vitro effects in many types of cancers and intracellular molecular targets modulated by this polyphenol. The authors provided an update of the clinical trials that suggested it to be a promising therapeutic and chemopreventive agent. 

Yang et al. reported adjuvants that enhance the anti-tumor effect and reduce the detrimental effect of Bleomycin (BLM) in mice [9]. The supercritical-carbon dioxide fluid extract from flowers and buds of *Chrysanthemum indicum* (CISCFE) exhibits potent anti-inflammatory, anti-oxidant, and lung protective effects. Their data suggest that the oral administration of CISCFE combined with BLM could markedly prolong the life span of a patient. In addition, it lessens many of the BLM-induced adverse effects. These results indicated that CISCFE could enhance the anti-cancer activity of BLM and reduce the BLM-induced pulmonary injury in tumor-bearing mice, rendering it as a potential adjuvant drug for BLM chemotherapy.

Mirzayans et al. provided an overview of the mechanisms by which *p53* signaling suppresses apoptosis following genotoxic stress, facilitating repair of genomic injury under physiological conditions but having the potential to promote tumor regrowth in response to cancer chemotherapy [10].

A case-control study of the genetic variability in reactive oxygen species (ROS)-metabolizing enzymes to risk of melanoma was reported by Yuan et al. [11]. They hypothesized that ROS producing and metabolizing enzymes were major contributors in UV-driven melanomas. In this study of 349 participants, the investigators genotyped 23 prioritized single nucleotide polymorphisms (SNPs) in nicotinamide adenine dinucleotide phosphate (NADPH) oxidases 1 and 4 (NOX1 and NOX4, respectively), CYBA, RAC1, superoxide dismutases (SOD1, SOD2, and SOD3) and catalase (CAT) and analyzed their associated melanoma risk. The results highlighted the importance of RAC1, SOD2, and SOD3 variants in the risk of melanoma.

To investigate the toxic mechanism of hexavalent chromium Cr(VI) and to search for an antidote for Cr(VI)-induced cytotoxicity, a study of mitochondrial dysfunction induced by Cr(VI) and cell survival by recovering mitochondrial function was performed by Zhong et al. [12]. They found that the gene expression of electron transfer flavoprotein dehydrogenase (ETFDH) was strongly downregulated by Cr(VI) exposure. The levels of coenzyme 10 (CoQ10) and mitochondrial biogenesis presented by mitochondrial mass and mitochondrial DNA copy number were also markedly reduced after Cr(VI) exposure. The Cr(VI)-induced harmful effects were lessened by pretreatment with CoQ10 in L-02 hepatocytes, which suggests that Cr(VI) induces CoQ10 deficiency in L-02 hepatocytes and that this deficiency may be a biomarker of mitochondrial dysfunction in Cr(VI) poisoning. Moreover, exogenous administration of CoQ10 may restore mitochondrial function and protect the liver from Cr(VI) exposure.

Liu et al. explored whether valproic acid (VPA, a histone deacetylase inhibitor) could radiosensitize osteosarcoma and primary-culture tumor cells and investigated the mechanism of VPA-induced radiosensitization [13]. They used osteosarcoma cells (U2OS) and primary-culture cells from chemical carcinogen 7,12-dimethylbenz[*a*]anthracene (DMBA)-induced breast cancer in rats. Clonogenic survival, immunofluorescence, fluorescent in situ hybridization (FISH) for chromosome aberrations, and comet assays were performed. The investigators found that VPA at the safe concentration (0.5 or 1.0 mM) could result in the accumulation of more ionizing radiation (IR)-induced DNA double strand breaks and increase the cell radiosensitivity. VPA-induced radiosensitivity was likely due to the inhibition of DNA repair activity. In addition, the chromosome aberrations including chromosome breaks, chromatid breaks, and radial structures were increased after the combination treatment of VPA and IR. The results obtained by primary-culture cells from the tissue of chemical carcinogen-induced breast cancer in rats also confirmed this observation. Therefore, VPA at a safe dose can act as a radiosensitizer for osteosarcoma and primary-culture tumor cells by suppressing DNA-double strand breaks repair.

The clinical impact of the expression of NOTCH1 signaling components in squamous cell carcinoma of the pharynx and larynx has been evaluated in subgroups by Wirth et al. [14] with the goal to evaluate NOTCH1 expression in head and neck squamous cell cancer (HNSCC) patient tissue and cell lines. The authors determined that NOTCH1 expression is associated with overall survival, but inhibition of NOTCH1 may not be a promising objective in HNSCC.

Povea-Cabello et al. investigated dynamic reorganization of the cytoskeleton during apoptosis [15]. Cells go through characteristic morphological changes during apoptosis and the cytoskeleton plays an active role. It appears that the apoptotic microtubule network (AMN) is essential to maintain plasma membrane integrity and cell morphology during the execution phase of apoptosis. The new “two coffins” hypothesis was offered, which postulates that both AMN and apoptotic cells can adopt two morphological patterns, round or irregular, resulting from different cytoskeleton kinetic reorganization during the execution phase of apoptosis induced by genotoxic agents. Moreover, round and irregular-shaped apoptosis implied different biological properties with respect to AMN maintenance, plasma membrane integrity, and phagocyte responses. These results suggest that characterizing the type of apoptosis may be important to anticipate how fast apoptotic cells undergo secondary necrosis and the subsequent immune response. In terms of pathology, round-shaped apoptosis can be considered a physiological and controlled type of apoptosis, while irregular-shaped apoptosis would be characterized as a pathological type of cell death closer to necrosis.

The variety of articles published in this Special Issue underscores the diversity of this field of research. This is only a snapshot of this very large area of research that includes thousands of active researchers working in many complementary directions to better understand the etiology of cancer and develop superior strategies for chemoprevention and cancer treatment. 
